# Predictive factors of quality of life among medical students: results from a multicentric study

**DOI:** 10.1186/s40359-021-00534-5

**Published:** 2021-02-25

**Authors:** Alice de Queiroz Constantino Miguel, Patricia Tempski, Renata Kobayasi, Fernanda B. Mayer, Milton A. Martins

**Affiliations:** 1 Centro de Desenvolvimento de Educação Médica, Faculdade de Medicina da Universidade de Sao Paulo, Av. Dr. Arnaldo 455 sala 1210, Sao Paulo, SP 01246-903 Brazil; 2 Departamento de Clínica Médica, Faculdade de Medicina da Universidade de São Paulo, São Paulo, Brazil; 3 Hospital Universitário da Universidade Federal de São Carlos, São Carlos, Brazil; 4 Pontifícia Universidade Católica do Paraná, Curitiba, Brazil

**Keywords:** Quality of life, Students, Medical, Time management, Predictive factors, Education, Medical, Multicenter study

## Abstract

**Background:**

Medical students have a worse perception of Quality of Life (QoL) and a high prevalence of psychosocial suffering when compared to the general population. The objective of this study was to investigate associated factors with Quality of Life of Brazilian medical students from an exploratory analysis in a cross-sectional study described in accordance with the STROBE (Strengthening the Reporting of Observational studies in Epidemiology) guidelines.

**Methods:**

This is a cross-sectional and multicenter study with national sample randomized by sex and year of the course. Data were collected between August 2011 and August 2012, using an electronic platform (VERAS platform). Our outcomes included: personal quality of life (QoLp) and quality of life related to medical course activities (QoLmc), both measured using a score ranging from 0 (worst) to 10 (best). Variables as predictors: the World Health Organization Quality of Life Assessment abbreviated version (WHOQOL-BREF); VERAS-Q (a questionnaire created to evaluate the QoL of students in health professions); Epworth Sleepiness Scale (ESS), Pittsburgh Sleep Quality Index (PSQI), Beck Depression Inventory (BDI), State-Trait Anxiety Inventory (STAI), Maslach Burnout Inventory (MBI), Resilience Scale (RS-14), Interpersonal Reactivity Multidimensional Scale (IRMS) and Dundee Ready Education Environment Measure (DREEM).

**Results:**

Our sample is comprised of 1350 (81.8%) medical students. When comparing predictors and both quality of life outcome measures, we found a negative correlation between QoL and the BDI, PSQI and ESS scores. Through a multiple linear regression mode we identified the median of significant predictors to higher QoL. We then run a tree regression model that demonstrated that the VERAS-Q—physical health domain (a domain assessing self-care, self-perception of health, sleep, leisure, physical activity and appearance) was the most important factor predicting QoL. Students with a VERAS-Q-physical health score ≥ 60.9 and a VERAS-Q-time management (address the management of the student's time, free time and whether he can dedicate himself to other activities besides the course) score ≥ 55.7 presented the best QoLmc (score: 8.08–9.63%). Students with a VERAS-Q-physical health score ≥ 79.7 presented the highest QoLp (score 8.93–8.74%).

**Conclusion:**

Physical symptoms, self-perception of health and self-care assessed by the VERAS-Q physical domain had association with both final outcomes. Time management seems to have a protective role for better Quality of Life. These variables should be taken in consideration when designing interventions to improve Quality of Life among medical students.

## Background

The stressful nature of medical education can affect medical student´s physical and mental health, and thus, their quality of life [1–7]. Medical students have worse perception of their Quality of Life (QoL) than the general population, even when matched by gender and age [8–12]. Worse perception of QoL among medical students was also observed comparing with other university students. Bittencourt and Paro [11] assessed the perception of QoL of students in Medicine, Nursing, Pharmacy and Speech Therapy courses. They observed that medical students had lower QoL scores compared to other students. Lack of free time and fatigue were considered the main reasons this difference [11].

In addition, they medical students present a high prevalence and intensity of psychosocial distress and mental disorders, such as depression, anxiety, burnout syndrome, suicidal ideation and attempt, pathological sleep disorders, alcohol and drug use and abuse [3, 4, 13–27]. This higher morbidity for mental disorders has a negative impact on academic and professional performance of these students [4, 28–32]

Humanistic theory from Carl Rogers states that people are always in the process of changing and growing. The ability to adapt, learn and change plays a vital role in this theory. Quality of life or happiness is a process, not a state of being. Using this framework, it is important to elucidate the factors that help and/or oppose this process in the life of medical students [33, 34].

Despite a growing number of studies on this topic, it is still not clear what are the risk factors associated to worse QoL as well as the protective factors. Few previous studies have globally investigated these factors. Notwithstanding, the identification of modifiable risk factors are relevant for the design of preventive interventions at an early stage in vulnerable individuals. Quality of life is a multidimensional construct with many protective and risk factors. In medical students it has been suggested that time management, physical activity, resilience and empathy can be protective and depression, anxiety, sleep deprivation and burnout may act in the opposite direction.

The aim of this research was to identify the factors that positively and negatively impact the quality of life of medical students. To accomplish it we made a multiple exploratory analysis to evaluate individual and medical curriculum related factors contributing toward higher and lower QoL levels among this group of individuals. For this analysis, we used data of a multicentric Brazilian study aimed to evaluate quality of life of medical students (VERAS an acronym that means Life of Students and Residents of Health Professions). We previously published data from this study showing associations between QoL and burnout, empathy, educational environment, leisure-time physical activity, resilience, anxiety and depression symptoms [35–40]. In the present study, we evaluate the influence of all these factors in QoL in general and QoL related to medical course QoL.

## Methods

This is a cross-sectional study to evaluate factors contributing toward Quality of Life among medical students. This study is described in accordance with the STROBE (Strengthening the Reporting of Observational studies in Epidemiology) guidelines [41].

The Institutional Review Board of the School of Medicine of the University of São Paulo (CEP-FMUSP number 181/11), as well as the institutional review boards of all other participating medical schools approved the study, with all participants providing informed consent before the implementation of any study protocol.

We conducted this investigation as part of a multicenter study involving 22 Brazilian medical schools (the VERAS study, translated to English as “Students’ and Residents’ life in health professions”). Detailed description of this study was previously published [2, 36–40, 42]. Schools participating in the study were from all regions of Brazil, and with a diverse legal status and location (13 public and 9 private); 13 in state capital cities and 9 in other cities). All medical schools included (Universidade Federal do Rio de Janeiro, Universidade Federal de Ciências da Saúde de Porto Alegre, Universidade Estadual do Piauí, Faculdade de Medicina de Petrópolis, Faculdade de Ciências Médicas da Paraíba, Pontifícia Universidade Católica de São Paulo, Universidade Federal do Ceará, Universidade Federal de Goiás, Universidade Federal de Mato Grosso do Sul, Escola Baiana de Medicina e Saúde Pública, Faculdade de Medicina de Marília, Faculdade de Medicina de São José do Rio Preto, Faculdade de Ciências Médicas da Paraíba, Faculdade Evangélica do Paraná, Faculdade de Medicina do ABC, Fundação Universidade Federal de Rondônia, Pontifícia Universidade Católica do Rio Grande do Sul, Universidade Federal do Tocantins, Universidade Federal de Uberlândia, Universidade Estadual Paulista Júlio de Mesquita Filho, Centro Universitário Serra dos Órgãos, Universidade de Fortaleza and Universidade de Passo Fundo[43]) approved the study.

Students were stratified into clusters by gender and program year, using a computer-generated list of random numbers. Medical students included in the study received a link from both institutional and personal emails to access an electronic survey platform designed specifically for this project. The first page of the survey was the written informed consent, the student could only continue if he/she read and agreed to participate in the study. Apart from data collected through formal, validated instruments (described below), we also collected socio-demographic data: age, sex, location and type of medical school (public or private), year of training education, information on financial support and housing, heigh and weight. Students were asked to complete the survey within 10 days. After completing the survey, students received automatic feedback on their scores for each domain of each questionnaire along with information about its interpretation. There were psychologists in the research group, so students could contact any of the coordinating researchers for guidance or emotional support throughout the study. Data were collected between August 2011 and August 2012.

### Participants

A total of 153 Brazilian medical schools were considered for inclusion in our study. We only included medical schools with at least one class in the process of graduation, bringing the total target population to approximately 86,000 medical students across all six years of training. We defined our sample size (n = 1152) to achieve an effect size of 0.165 comparing two groups with the same size, also assuming a statistical power of 80% and a 0.05 significance level. We later increased the sample to 1650 students to account for a 30% loss of participants. We then randomly selected at least 60 students from each of 22 medical schools. Next, we stratified all participants into clusters by gender and program year, i.e., five males and five females per each of the six program years, using a computer-generated list of random numbers. We excluded students who have not answer 100% of the questionnaires. To avoid sampling bias due to a low percentage of responses, we defined a minimum response rate of 60% in each medical school to include the responses in the final analysis, according to the recommendation of Huston [44].

### Outcomes

Our outcomes of interest included: (a) personal quality of life (QoLp) measured using a self-reported analog scale with a score ranging from 0 (worst) to 10 (best), and (b) quality of life (QoLmc) related to medical course activities measured using a score ranging from 0 (worst) to 10 (best).

### Predictors

We selected the following scales and instruments to investigate predictive factors:

*World Health Organization Quality of Life Assessment abbreviated version (WHOQOL-BREF)* is a brief version of the WHOQOL 100 and consists of 26 items, clustered into the following four domains: physical health, psychological health, social relationships and environment. Higher scores represent better QoL. This questionnaire was translated and validated to Brazilian Portuguese [45, 46]. The Cronbach´s alpha for each domain was 0.66 for social relationships, 0.73 for environment, 0.75 for physical health and 0.79 for psychological health.

*VERAS-Q* is a questionnaire created to evaluate the QoL of students in health professions (Additional file 1). It consists of 45 items with a 5-point Likert scale divided into four domains (time management, psychological, physical health, and learning environment) and a global score. The score ranges from 0 (worst quality of life) to 100 (best quality of life) [47]. The Cronbach´s alpha for each domain was 0.77 learning environment, 0.79 for physical health, 0.82 for both psychological and time management and 0.91 for global score.

*Epworth Sleepiness Scale (ESS)* is a self-reported questionnaire evaluating the likelihood of falling asleep in eight situations involving daily activities, on a scale of 0–3 (where 0 = would never doze and 3 = high chances of dozing). The overall score ranges from 0 to 24 with higher scores representing a person's daytime sleepiness. A version has been translated and validated for Brazilian Portuguese [48, 49]. The Cronbach´s alpha for this questionnaire was 0.74.

*Pittsburgh Sleep Quality Index (PSQI)* measures self-reported sleep quality and disturbance over the past one month. The PSQI is composed of 19 items, which are combined into seven constructs that are summarized into a global score ranging from 0 to 21, where the highest score indicates worst sleep quality. This questionnaire was translated and validated to Brazilian Portuguese [50, 51]. The Cronbach´s alpha for this questionnaire was 0.65.

*Beck Depression Inventory (BDI),* is a 21-item, self-reported measure that measures characteristic attitudes and symptoms of depression. Responses are scored on a 4-point scale, ranging from 0 to 3, and total scores can range from 0 to 63. The higher the score, the greater the symptom intensity. This questionnaire was translated and validated to Brazilian Portuguese [52, 53]. The Cronbach´s alpha for this questionnaire was 0.87.

*Resilience Scale (RS-14)* measures the level of individual resilience, a person's ability to cope with problems and withstand pressure in adverse situations without suffering physical, psychological, or social harm. It involves adaptive skills, flexibility, interaction, overcoming and resignification of lived experiences. The scale consists of 14-items and was translated and validated to Brazilian Portuguese [54, 55]. The Cronbach´s alpha for this questionnaire was 0.87.

*State-Trait Anxiety Inventory (STAI)* is a self-reported questionnaire consisting of 40 items used to measure the presence and severity of anxiety symptoms. This scale is composed of two distinct subscales: the state anxiety scale evaluates current behavior while the trait anxiety scale evaluates personality. This questionnaire was translated and validated to Brazilian Portuguese [56–58]. The Cronbach´s alpha for this questionnaire was 0.92 and 0.93, respectively for Trait and State anxiety.

*Interpersonal Reactivity Multidimensional Scale (IRMS)* evaluates empathy and its associated factors. It consists of 21 statements describing personal characteristics and following three main domains including empathic, emotional, and behavioral components. Medical students' empathy was measured using three subscales of the validated version of the EMRI for Brazilian-Portuguese [59, 60]. The Cronbach´s alpha for each domain was 0.61 for emotional, 0.73 for empathic and 0.77 for behavioral.

*Maslach Burnout Inventory (MBI)* is a burnout assessment instrument consisting of 22 items divided into three domains: emotional exhaustion, depersonalization, and personal accomplishment. Each domain is scored on a seven-point Likert scale, with values from 0 (never) to 6 (every day). Burnout intensity is determined by the summing the scores of questions pertaining to each domain. Higher scores correspond to a higher degree of burnout. This questionnaire was translated and validated to Brazilian Portuguese [61, 62]. The Cronbach´s alpha for each domain was 0.68 for depersonalization, 0.81 for personal accomplishment and 0.85 for emotional exhaustion.

*Dundee Ready Education Environment Measure (DREEM)* contains 50 statements relating to the educational environment, scored on a five-point Likert scale (0 = strongly disagree and 4 = strongly agrees), some of which are inverted. Higher scores indicate a positive evaluation. The DREEM has a maximum score of 200, which represents an ideal educational environment. The subscales are as follows: Students' perceptions of Learning (SPL); Students' perceptions of Teachers (SPT; Students' Academic Self Perception (SASP); Students' perceptions of Atmosphere (SPA); Students' social self-perceptions (SSSP). This questionnaire was translated and validated to Brazilian Portuguese [63, 64]. The Cronbach´s alpha for each domain was 0.65 for SSSP, 0.71 for SASP, 0.82 for SPL, 0.83 for SPA, 0.84 for SPT and 0.94 for global score.

### Potential confounders

We selected potential confounders using a combination of clinical judgment and evidence from the literature, as these joint criteria have been demonstrated to perform better than the isolated selection of isolated clinical or evidence-based criteria [65]. We selected age, gender, body mass index, and school levels as potential confounders [66].

### Statistical analysis

We started with a descriptive and visual exploratory analysis of all variables to evaluate their frequency, percentage and near-zero variance for categorical variables, distribution for numeric variables, and their corresponding missing value patterns [67]. Comparisons for the exploratory analysis were conducted through analysis of variance (*t*-tests being a category of analysis of variance) and Chi-square tests (Fisher exact test when any cell presented a frequency below 5).

Our strategy to evaluate predictors and their relationship with study outcomes (personal and related to medical course QoL) made use of a series of generalized linear models with a Gaussian family, i.e., Multiple Linear Regression Models (MLRM). The regression models were adjusted for age, gender, body mass index, and school levels. We used p for trend to measure the impact of individual numeric predictors on each of the QoL outcomes. In order to calculate predicted mean values for the outcome rather than simply obtaining less clinically interpretable measures of correlation, we categorized predictors at their median value (for example, median of Resilience Scale scores of 81, global DREEM score of 120). Results are reported as predicted means with 95% confidence intervals, with results being interpreted as significant when the confidence intervals do not overlap between different estimates along with p for trend < 0.001 [68].

We also used Regression Trees Model (RTM) with the same set of previously described outcomes and predictors. Regression trees complement the use of machine learning models as they represent the best cut-points for predictor values in the context of a given outcome after previous predictors have been taken into account. In order to avoid overfitting, we used a cost-complexity pruning strategy using the weakest link pruning strategy by successively collapsing the internal node that produces the smallest per-node increase in the cost complexity criterion [69]. When overfitting is detected, those nodes were removed. Otherwise, they were left intact. We have also provided a graphical representation of each model.

All analyses were performed using the R language with packages ggplot2 and rmarkdown; using the SPSS software version 22;

## Results

### Descriptive and visual exploratory analysis

Table 1 displays the description of the overall study sample. Our sample is comprised of 1350 medical students, most of them being females (52.9%), the sample having a mean age of 22.8 years and an average Body Mass Index of 23.2 ± 3.5 kg/m^2^.

**Table 1:** Sample characteristics

Variable	Total (SD)
	1350
Age (years)	22.8 (± 3.01)
BMI (kg/m^2^)	23.2 (± 3.46)
Schooling level (years of medical school)	
1–2	459 (34.0%)
3–4	491 (36.4%)
5–6	400 (29.6%)
QoL personal	7.86 (± 1.27)
QoL medical course	6.51 (± 1.56)
WHO QOL	
Physical health	65.2 (± 14.7)
Psychological	61.7 (± 15.7)
Social relations	63.6 (± 19.9)
Environment	63.8 (± 14.1)
VERAS-Q	
Time management	37.4 (± 15.5)
Psychological	51 (± 16.1)
Physical health	54.5 (± 18.3)
Learning environment	57.2 (± 13.1)
Epworth sleepiness scale	10.3 (± 3.9)
Global PSQI	6.72 (± 3.02)
Becks depression inventory	9.37 (± 7)
Resilience scale (RS-14)	78.7 (± 12.4)
STAI	
State	43.7 (± 11.6)
Trait	45.5 (± 11.7)
IRMS	
Empathy	26.1 (± 4.82)
Emotional	24.7 (± 5.04)
Personal anguish	19.2 (± 4.22)
MBI	
Emotional exhaustion	26.7 (± 9.8)
Depersonalization	8.55 (± 5.74)
Reduced personal accomplishment	33.8 (± 7.61)
Global DREEM* score	119 (± 27.1)

*SD* standard deviation, *BMI* Body Mass Index, *BDI *beck depression inventory, *PSQI* Pittsburgh Sleep Quality Index, *STAI *state-trait anxiety inventory, *IRMS *interpersonal reactivity multidimensional scale, *MBI *Maslach burnout inventory, *DREEM *Dundee ready education environment measure, *RS-14 *resilience scale

### Univariate analysis

**Fig. 1:**
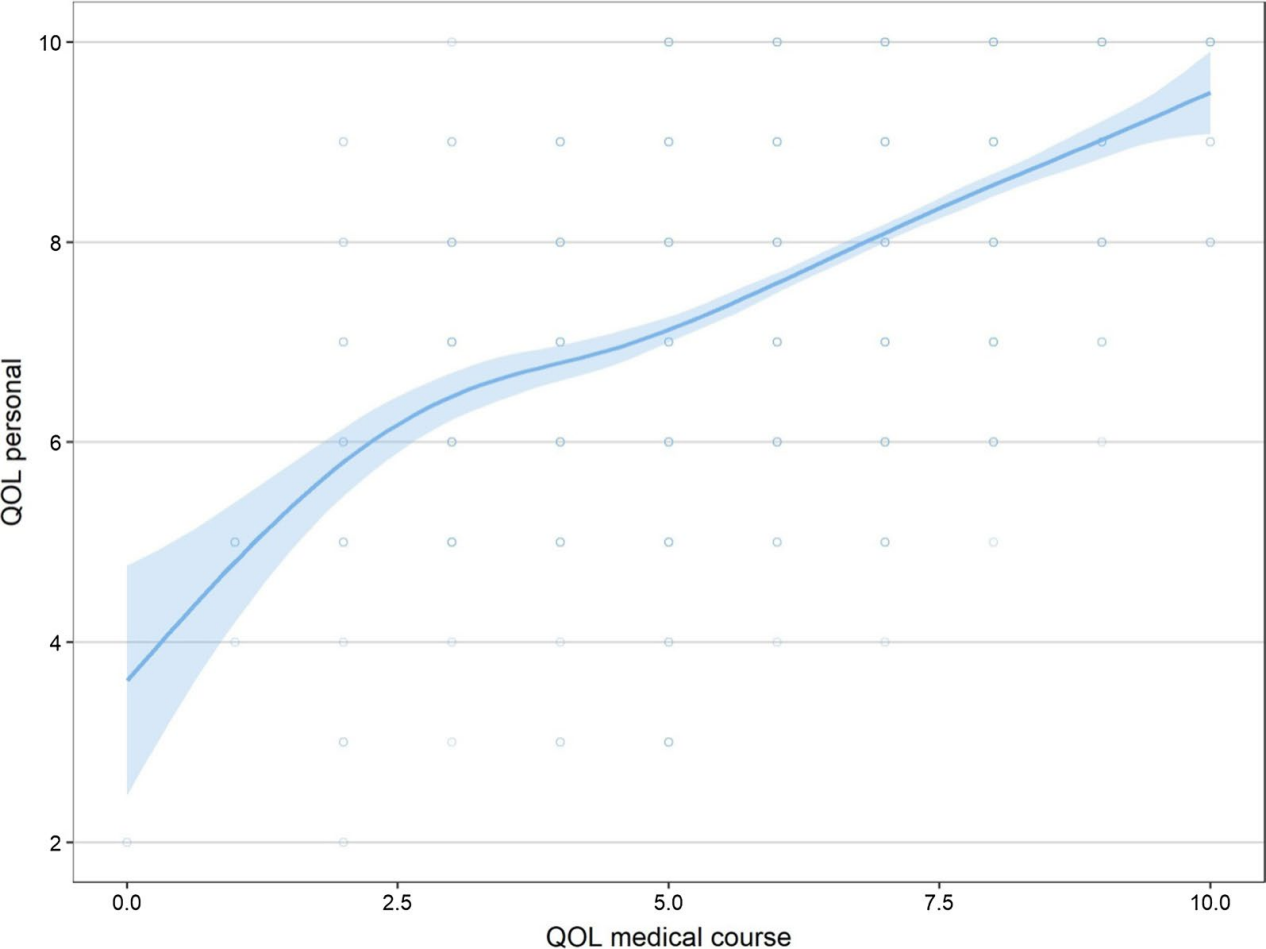
Association between personal and medical course quality of life. Legend: QOL personal–personal Quality of Life; QOL medical course-Quality of Life related to medical course

When evaluating the association between study outcomes, personal QoL and QoL related to medical course presented a positive correlation (0.566; *p* < 0.001) (Fig. 1).

When comparing predictors and both quality of life outcome measures, we found a negative correlation between QoL related to medical course and the Beck Depression Inventory (correlation = − 0.397, *p* < 0.001), Pittsburgh Sleep Quality Index (0.345, *p* < 0.001) and Epworth Sleepiness Scale scores (− 0.211, *p* < 0.001). These finding indicate that an increased quality of life is associated with lower depression symptoms, better sleep quality and lower levels of daytime sleepiness. Correlation with the same direction was observed between personal QoL and Beck Depression Inventory (− 0.339, *p* < 0.001), Pittsburgh Sleep Quality Index (− 0.276, *p* < 0.001) and the Epworth Sleepiness Scale scores (− 0.092, *p* < 0.001).

### Multiple linear regression model (MLRM)

Table 2 shows the association between predictors and both QoL related to medical course and personal QoL, evaluated through a multiple linear regression model and adjusted for age and Body Index Mass.

**Table 2:** Predictors of quality of life of medical students adjusted for age and BMI

Predictor (median)	QOL medical course	*p* for trend	QOL personal	*p* for trend
	Mean (CI)		Mean (CI)	
Beck depression inventory		*p* < 0.001		*p* < 0.001
≤ 8	6.96 (6.85, 7.06)		8.17 (8.08, 8.26)	
> 8	6.02 (5.90, 6.13)		7.53 (7.43, 7.62)	
Global PSQI		*p* < 0.001		*p* < 0.001
≤ 6	6.93 (6.82, 7.04)		8.12 (8.02, 8.21)	
> 6	6.07 (5.95, 6.18)		7.60 (7.50, 7.69)	
Epworth sleepiness scale		*p* < 0.001		*p* < 0.001
≤ 10	6.84 (6.72, 6.95)		7.98 (7.89, 8.08)	
> 10	6.14 (6.02, 6.26)		7.73 (7.63, 7.83)	
WHO QOL—environment		*p* < 0.001		*p* < 0.001
≤ 65.6	6.04 (5.94, 6.14)		7.49 (7.41, 7.57)	
> 65.6	7.14 (7.02, 7.26)		8.36 (8.27, 8.46)	
WHO QOL—social relations		*p* < 0.001		*p* < 0.001
≤ 66.7	6.22 (6.11, 6.32)		7.61 (7.52, 7.7)	
> 66.7	6.94 (6.81, 7.06)		8.23 (8.13, 8.33)	
WHO QOL—psychological		*p* < 0.001		*p* < 0.001
≤ 62.5	5.98 (5.87, 6.08)		7.48 (7.4, 7.57)	
> 62.5	7.13 (7.01, 7.24)		8.30 (8.21, 8.40)	
WHO QOL—physical health		*p* < 0.001		*p* < 0.001
≤ 67.9	6.06 (5.96, 6.16)		7.58 (7.5, 7.66)	
> 67.9	7.19 (7.07, 7.31)		8.29 (8.19, 8.39)	
STAI—state		*p* < 0.001		*p* < 0.001
≤ 43	6.98 (6.87, 7.09)		8.23 (8.14, 8.32)	
> 43	6.02 (5.91, 6.14)		7.48 (7.39, 7.57)	
STAI—trait		*p* < 0.001		*p* < 0.001
≤ 45	7.00 (6.89, 7.11)		8.23 (8.14, 8.32)	
> 45	6.01 (5.9, 6.13)		7.49 (7.39, 7.58)	
EMRI/IRMS—empathy		*p* = 0.361		*p* = 0.972
≤ 26	6.55 (6.43, 6.67)		7.87 (7.77, 7.96)	
> 26	6.47 (6.35, 6.59)		7.86 (7.77, 7.96)	
EMRI/IRMS—emotional		*p* = 0.298		*p* = 0.887
≤ 25	6.47 (6.36, 6.59)		7.87 (7.78, 7.96)	
> 25	6.56 (6.44, 6.68)		7.86 (7.76, 7.96)	
EMRI/IRMS—personal anguish		*p* < 0.001		*p* < 0.001
≤ 19	6.65 (6.54, 6.76)		8.00 (7.91, 8.09)	
> 19	6.35 (6.22, 6.47)		7.70 (7.60, 7.80)	
MBI—emotional exhaustion		*p* < 0.001		*p* < 0.001
≤ 27	7.02 (6.92, 7.13)		8.13 (8.04, 8.22)	
> 27	5.94 (5.83, 6.05)		7.57 (7.47, 7.66)	
MBI—depersonalization		*p* < 0.001		*p* < 0.001
≤ 8	6.78 (6.67, 6.9)		8.01 (7.92, 8.11)	
> 8	6.19 (6.07, 6.31)		7.69 (7.59, 7.79)	
MBI—reduced personal accomplishment		*p* < 0.001		*p* < 0.001
≤ 35	6.24 (6.12, 6.35)		7.68 (7.58, 7.77)	
> 35	6.84 (6.72, 6.96)		8.09 (7.99, 8.19)	
VERAS-Q—time management		*p* < 0.001		*p* < 0.001
≤ 36.4	5.95 (5.85, 6.06)		7.62 (7.53, 7.71)	
> 36.4	7.15 (7.04, 7.26)		8.14 (8.04, 8.24)	
VERAS-Q—psychological		*p* < 0.001		*p* < 0.001
≤ 50	5.98 (5.87, 6.09)		7.49 (7.4, 7.58)	
> 50	7.07 (6.96, 7.18)		8.25 (8.16, 8.35)	
VERAS-Q—physical health		*p* < 0.001		*p* < 0.001
≤ 53.1	5.90 (5.79, 6.01)		7.34 (7.25, 7.43)	
> 53.1	7.14 (7.03, 7.25)		8.4 (8.32, 8.49)	
VERAS-Q—learning environment		*p* < 0.001		*p* < 0.001
≤ 57.1	5.94 (5.83, 6.05)		7.54 (7.45, 7.63)	
> 57.1	7.12 (7.01, 7.23)		8.21 (8.11, 8.30)	
Global DREEM score		*p* < 0.001		*p* < 0.001
≤ 120	6.00 (5.89, 6.11)		7.63 (7.53, 7.72)	
> 120	7.05 (6.93, 7.16)		8.11 (8.02, 8.21)	
RS-14 score		*p* < 0.001		*p* < 0.001
≤ 81	6.24 (6.13, 6.35)		7.61 (7.52, 7.7)	
> 81	6.82 (6.7, 6.94)		8.15 (8.05, 8.25)	

*CI* confidence interval, *PSQI* Pittsburgh Sleep Quality Index, *STAI *state-trait anxiety inventory, *IRMS *Interpersonal Reactivity Multidimensional Scale, *MBI *Maslach burnout inventory, *DREEM *Dundee ready education environment measure, *RS-14 *resilience scale

When comparing predictors with both QoL related to medical course and personal QoL, significant predictors of higher QoL included: Beck Depression Inventory (above and below a median of 8) (6.96 vs. 6.02 for QoL medical course, 8.17 vs. 7.53 for QoL personal), global Pittsburgh Sleep Quality Index at median of 6 (6.93 vs. 6.07, 8.12 vs. 7.60), Epworth Sleepiness Scale at median 10 (6.84 vs. 6.14, 7.98 vs. 7.73), WHOQOL-BREF domains scores including environment at median of 65.6 (6.04 vs. 7.14, 7.49 vs. 8.36), social relations at median of 66.7 (6.22 vs. 6.94, 7.61 vs. 8.23), psychological at median of 62.5 (5.98 vs. 7.13, 7.48 vs. 8.30), and physical health at median of 67.9 (6.06 vs. 7.19, 7.58 vs. 8.29), VERAS-Q domains including time management scores at median of 36.4 (5.95 vs. 7.15, 7.62 vs. 8.14), psychological at median of 50 (5.98 vs. 7.07, 7.49 vs. 8.25), physical health at median 53.1 (5.90 vs. 7.14, 7.34 vs. 8.40), and learning environment at median of 57.1 (5.94 vs. 7.12, 7.54 vs. 8.21), State-Trait Anxiety Inventory—State score at median 43 (6.98 vs. 6.02, 8.23 vs. 7.48), State-Trait Anxiety Inventory—Trait score at median 45 (7 vs. 6.01, 8.23 vs. 7.49), Interpersonal Reactivity Multidimensional Scale-Personal Anguish domain score at median of 19 (6.65 vs. 6.35, 8.00 vs. 7.70), Maslach Burnout Inventory domains including emotional exhaustion at median of 27 (7.02 vs. 5.94, 8.13 vs. 7.57), depersonalization with median of 8 (6.78 vs. 6.19, 8.01 vs. 7.69), and personal accomplishment with median of 35 (6.24 vs. 6.84, 7.68 vs. 8.09), Dundee Ready Education Environment Measure global score at median of 120 (6.00 vs. 7.05, 7.63 vs. 8.11), and Resilience Scale score at median of 81 (6.24 vs. 6.82, 7.61 vs. 8.15), all demonstrating non-overlapping confidence intervals in relation to their predicted means with a *p* for trend < 0.001.

### Tree regression model

**Fig. 2:**
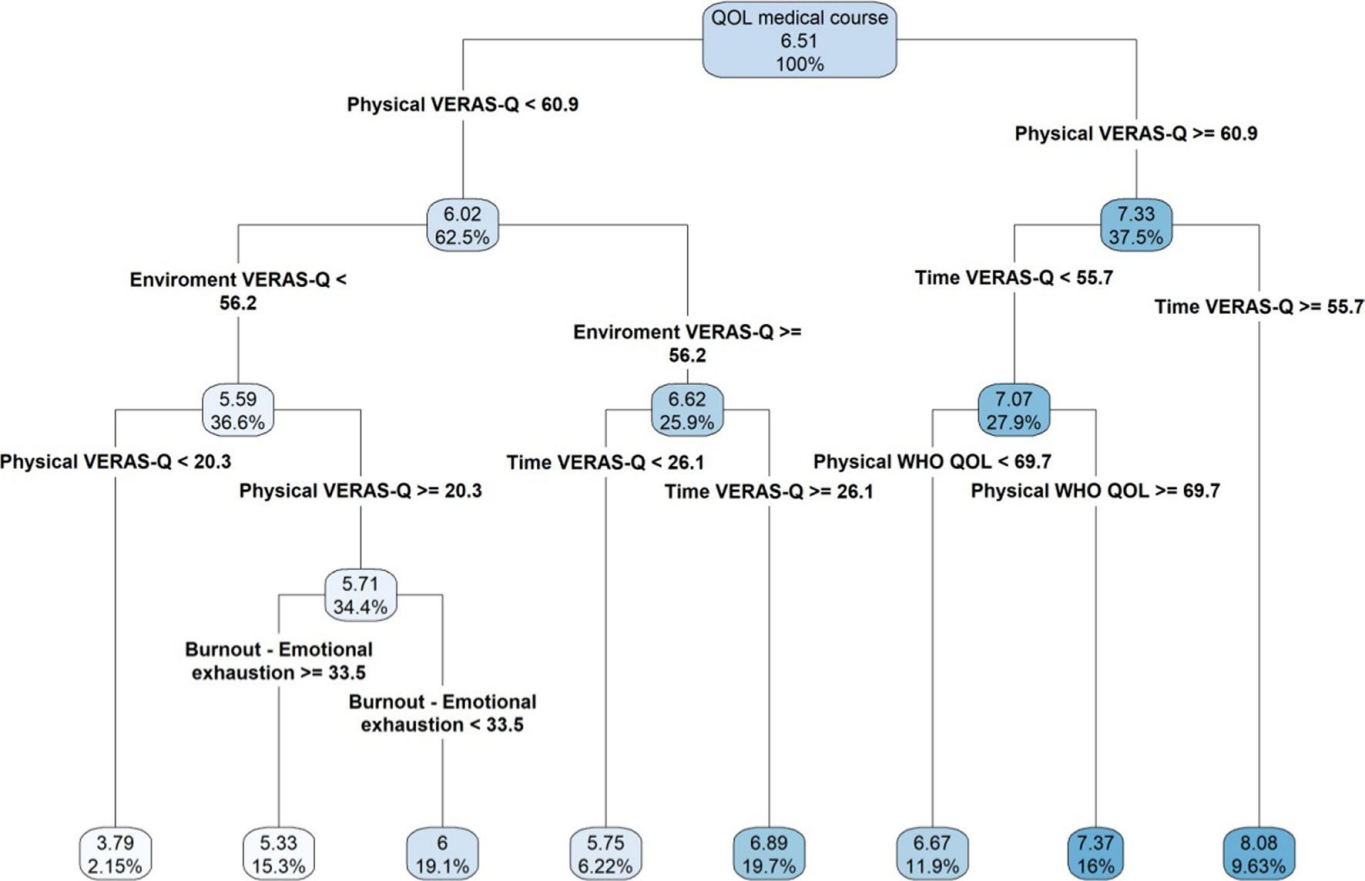
Regression tree of quality of life related to medical course. Legend: QOL medical course-Quality of Life related to medical course

We then attempted to validate our results by running a tree regression model (recursive partitioning) including all questionnaires and domain as predictors, identifying the main sequential factors leading to the prediction of medical student’s quality of life related (QoL) to the medical course. The model demonstrated that the VERAS-Q—physical health domain was the most important factor predicting QoL, followed by VERAS-Q-time management, VERAS-Q-learning environment, WHOQOL-BREF-physical health, and Maslach Burnout Inventory-Emotional exhaustion domain. We found that the students with a VERAS-Q-physical health score ≥ 60.9 and a VERAS-Q-time management score ≥ 55.7 presented the best QoL related to the medical course with a score of 8.08 (9.63%), while those with VERAS-Q-physical health score < 60.9 associated with a VERAS-Q-learning environment score < 56.2 were associated with the lowest QoLmc (3.79, 2.15%). Additional combinations of scores were associated with intermediate QoLmc scores as demonstrated in Fig. 2.

**Fig. 3:**
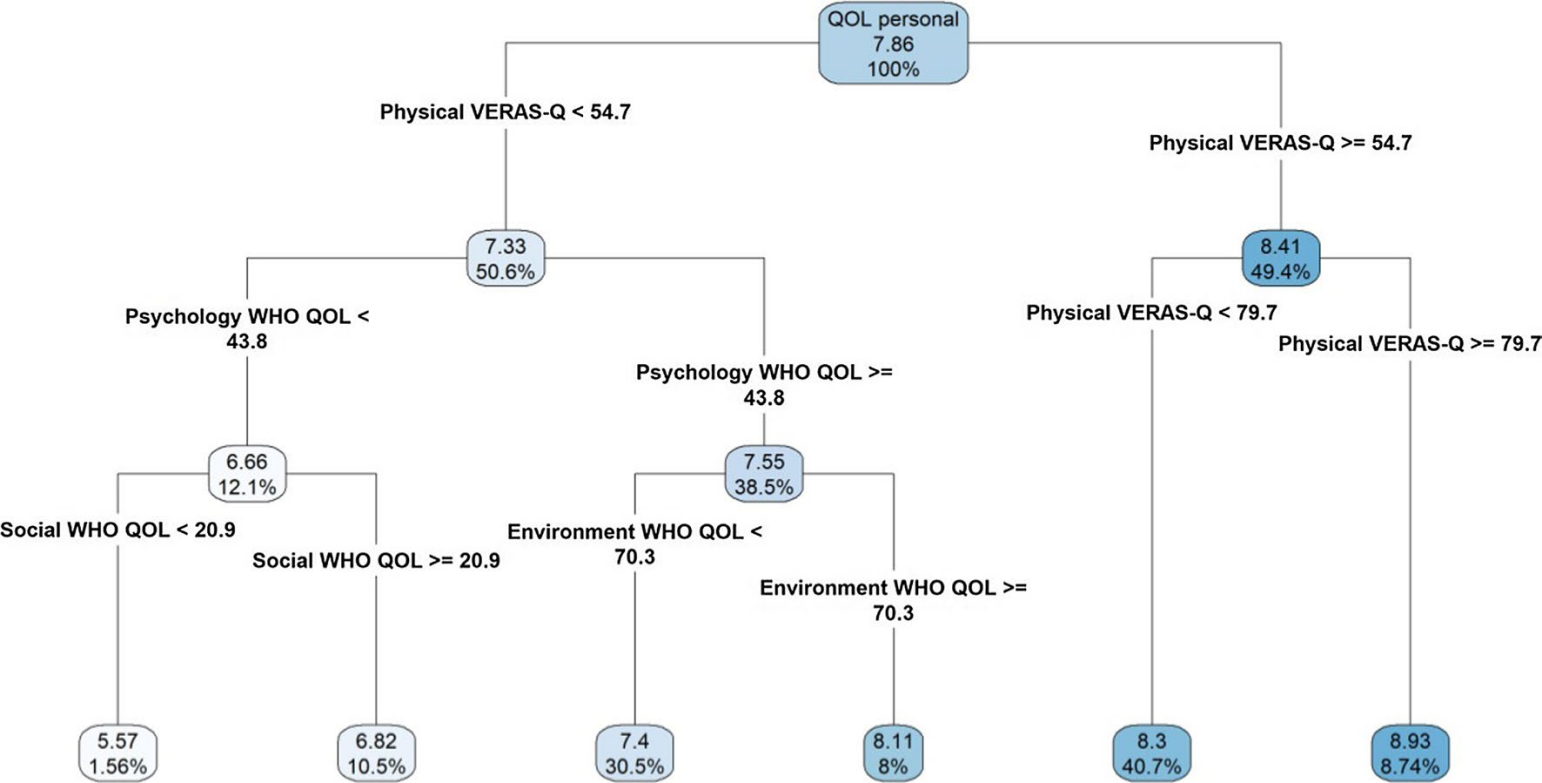
Regression tree of personal quality of life. Legend: QOL personal–personal Quality of Life

When evaluating all predictors of student’s personal QoL, the VERAS-Q-physical health domain was the most significant predictor, followed by WHOQOL-BREF-psychological health, WHOQOL-BREF-environment, and WHOQOL-BREF-social relationships domains. Students with a VERAS-Q-physical health score ≥ 54.7 presented the highest personal QoL score of 8.93 (8.74%), whereas the lowest QoLp score (5.57, 1.56%) was found among students with a VERAS-Q-physical health score of < 54.7 associated with a WHOQOL-psychological health score < 43.8 and a WHOQOL-social relationships score < 20.9. Additional combinations of scores were associated with intermediate personal QoL scores as demonstrated in Fig. 3.

## Discussion

In this study, we chose to explore the characteristics of the medical student's Quality of Life in an innovative and global way. We did so by seeking mainly protective factors of the educational environment and the medical training program, which can be an effective strategy to reverse stigma associated with the mental health of these students. In addition, by expanding knowledge about associated factors to mental health protection for medical students, the data can provide a substrate for the continuous improvement of medical school curricula.

The general sample of this study showed a discreet predominance of females (52.9%), reflecting a current international and national trend of “feminization” of medicine [70–72]. In Brazil, since 2005, there are more women in medical school than men and in 2011–2012, when this study was carried out, females represented 54.3% of the medical students [70]. In addition, the sample consists of young people with an average age of 22.8 years.

Analyzing the outcomes: personal and medical course Quality of Life (QoLp and QoLmc respectively), we noticed that there is a positive and concordant correlation between them. Medical students, however, attributed worse scores to QoLmc. In the data collection, these two questions were triggered consecutively, and their answers were scored from 0 to 10, making clear the minimum and maximum values. Therefore, it looks like that it was intentional on the part of the students to reinforce this difference, giving indications that the teaching environment and the curricular requirements directly affect their perception of Quality of Life and, thus, expressing their discontent. Several authors have already pointed out the dissatisfaction of medical students with the course and the direct negative influence on their Quality of Life, physical and mental health [1, 2, 73, 74].

In the general analysis of the questionnaires assessing Quality of Life (WHOQOL-BREF and VERAS-Q) the most visually significant result was the mean of the VERAS-Q time management domain (37.4), which is much lower than in the other domains of the same questionnaire (from 51 to 57). This was repeated in the analysis made by multiple linear regression. The univariate inferential analysis showed a negative correlation between depressive symptoms, daytime sleepiness and sleep disorder with the Quality of Life outcomes of this study (QoL personal and in medical course). When interpreting the results of multiple linear regression based on the questionnaire cuts, we identified that any degree of depressive symptom, mild to severe, is associated with worse QoLp and QoLmc (IDB > 8). Likewise, any degree of daytime sleepiness and poor sleep quality is associated with worse QoL personal and in medical course (Epworth > 10 and PSQI > 6). We understand that depressive symptoms, poor sleep quality and daytime sleepiness have a negative impact and are risk factors for a worse Quality of Life.

This result presents clinical and physiological corroboration, in addition to several other studies with similar conclusions: studies attribute to the medical course a negative effect on the mental health of medical students, resulting in a high prevalence of depression, anxiety, stress and sleep disorders [18–20, 37, 75–77]. In addition, sleep disorders lead to mental, psychological and physical morbidities [78–80].

Students with moderately high and high resilience had better perceptions of personal and medical course QoL (RS-14 > 81), suggesting it was a protective factor. This result is in line with other studies that demonstrate a dose–effect relationship between resilience, perception of the teaching environment and quality of life for medical students [35, 42, 81–83]. Accordingly, there is a current international movement for valuing this characteristic that has even become part of the new essential competences for the newcomer by the AAMC (Association of American Medical Colleges) [83].

Likewise, a better perception of the teaching environment (Excellent and More positive than negative in DREEM questionnaire) was also associated with better Quality of Life, as in other publications, but it is difficult to verify the causal relationship between them [40].

Regarding the findings obtained by the tree regression, our first conclusion is that VERAS-Q questionnaire developed for evaluation Quality of Life in medical students had greater power to discriminate students with better and worse quality of life in the course of medicine. Another interesting evidence was the secondary role of the results of the other questionnaires (MBI, BDI, IDATE, RS-14, EMRI, Epworth, PSQI and DREEM) and, therefore, did not enter the tree regression. In the literature, we realized that most studies focus on the assessment of students' physical, psychological and mental health through these questionnaires.

The first tree node in both personal and in the course QoL tree regression, was the Physical domain of VERAS-Q. This domain assesses self-care, self-perception of health, sleep, leisure, physical activity and appearance. That is, aspects that may reflect characteristics raised as risk and protective factors (depressive and anxious symptoms, burnout, sleep disorders and physical activity) and a global assessment (that mirrors this information indirectly and its physical impact) played a predominant role. This may reflect the multidimensional and subjective definition of Quality of Life and that, according to WHO, health and well-being are not just the absence of disease [84, 85]

When we focus on the personal Quality of Life (QoLp), the group with the best perception of physical health (score in the Physical domain of VERAS-Q > 54.7) had the best scores in the QoLp, and those with scores greater than 79.7 had an average of QoLp at 8.93. Remembering that the value associated with better or worse Qolp by multiple linear regression was 53.1. Thus, we realized that our two analyzes found similar results, and therefore, better perception of health and self-care, in addition to sleep, leisure, physical activity and appearance, seems to be protective factors.

Leaving to the other branch of the tree towards students with worse QoLp (average of 5.57), they score less than 54.7 in the Physical domain of VERAS-Q, less than 43.8 in the Psychological domain of WHOQOL-BREF, and less than 20.9 in the WHOQOL-BREF Social Relations domain. The Psychological domain of WHOQOL-BREF addresses Quality of Life in relation to positive thoughts, thinking, learning, memory, concentration, self-esteem, body image, negative feelings, spirituality and personal beliefs; and the WHOQOL-BREF Social Relations domain evaluates interpersonal and social relationships, support networks and sexual activity. The fact that the sequence of these domains (Physical of VERAS-Q, Psychological and Social Relations of WHOQOL-BREF) is more significant to characterize students with worse grades of QoLp (to the detriment of the other questionnaires), demonstrates the reliability of the WHOQOL-BREF questionnaire and confirms the multidimensionality of the Quality of Life concept [86].

The Quality of Life tree regression in the medical course (QoLmc) also begins in the Physical domain of VERAS-Q, but at a higher value than in the QoLp regression tree (60.9 × 54.7). This difference can be explained by the academic requirements and therefore, lower degrees of worse perception of physical health already have an impact on satisfaction with the course.

In the branch of students with better QoLmc, the time management domain of VERAS-Q above 55.7 appears as the second determinant. This value being well above the average found in the sample and the dividing value in the multiple linear regression (37.4 and 36.4). In this domain the questions address the management of the student's time, free time and whether he can dedicate himself to other activities besides the course.

Moving to the other extreme, the worst QoLmc scores (mean of 3.79) are associated with VERAS-Q Physical domain scores (< 20.3) and VERAS-Q Teaching Environment scores less than 56.2, a value similar to that found in multiple linear regression (57). This domain covers the teaching environment, the organization of the course, relations with colleagues, teachers and the educational institution. Therefore, students with the worst perceptions of physical health and self-care, as well as the worst perception of the teaching environment, make up the group of worst perceptions of Quality of Life in the medical course.

This study has important strengths. We randomly selected our sample to reduce response bias and included 22 medical schools, representing every region of Brazil, allowing us to generalize our results to medical students throughout the country. However, it has some limitations. The main limitation is its cross-sectional design that prevents us from making causal inferences. Another potential limitation is that some instruments had a Cronbach's alpha < 0.70. In addition, we cannot directly generalize our results to other countries.

## Conclusion

Assessing the medical student's Quality of Life globally, the specific symptoms of psychosocial suffering and mental disorders (such as depression, anxiety, burnout and sleep) individually have less association with the final outcome than a general perception of students regarding their physical and mental health, both for better and for worse Quality of Life. A questionnaire specifically developed for evaluation Quality of Life in medical students had greater power to discriminate students with better and worse quality of life in the course of medicine.

Another striking result of our analyzes is the protective role of the time management variable. Time management is considered the key to success in several professions and is also necessary for the balance of personal and professional life. Thus, we understand that educational institutions, in addition to revising their curricula to include free time for study and leisure, should introduce activities for students to develop time management skills.

## Supplementary Information

**Additional file 1:** VERAS-Q questionnaire.

## Data Availability

The datasets used and/or analyzed during the current study are available from the corresponding author on reasonable request.

## Abbreviations

AAMC: Association of American Medical Colleges
BDI: Beck depression inventory
BMI: Body Mass Index
DREEM: Dundee ready education environment measure
SD: Standard deviation
ESS: Epworth sleepiness scale
IRMS: Interpersonal reactivity multidimensional scale
MBI: Maslach burnout inventory
MLRM: Multiple linear regression models
PSQI: Pittsburgh sleep quality index
QoL: Quality of life
QoLp: Personal quality of life
QoLmc: Quality of life related to medical course activities
RS-14: Resilience scale
RTM: Regression trees model
SPSS: Statistical product and service solutions
STAI: State-trait anxiety inventory
STROBE: Strengthening the reporting of observational studies in epidemiology
VERAS: An acronym that means life of students and residents of health professions
VERAS-Q: An acronym that means life of students and residents of health professions questionnaire
WHO: World Health Organization
WHOQOL-BREF: The World Health Organization Quality of Life Assessment abbreviated version
