# Supplementary material for: Predictive factors of quality of life among medical students: results from a multicentric study
Source: BMC Psychol. 2021 Feb 25;9:36. doi: 10.1186/s40359-021-00534-5 (PMC7905855; doi:10.1186/s40359-021-00534-5)
Supplement: Supplementary file 1 — Additional file 1: VERAS-Q questionnaire. [file 40359_2021_534_MOESM1_ESM.docx]

**Appendix – Questionnaire to evaluate quality of life in medical students (VERAS-Q questionnaire)**

Answer according to your values, aspirations, worries and satisfactions of the past two weeks. Mark down one of the alternatives below:

TD – totally disagree D – disagree I – indifferent A – agree TA – totally agree

*Domains: TM-time management; P-psychological; PH- physical health; LE-learning environment.*

| Question |  |  |  |  |  |  |
| --- | --- | --- | --- | --- | --- | --- |
| 01. My quality of life is good. | *PH* | TD | D | I | A | TA |
| 02. I don’t make the most of my life. | *TM* | TD | D | I | A | TA |
| 03. I get supervision in my practice. | *LE* | TD | D | I | A | TA |
| 04. I have time for my family. | *TM* | TD | D | I | A | TA |
| 05. Sometimes I feel humiliated in the medical course. | *LE* | TD | D | I | A | TA |
| 06. My university environment is health. | *LE* | TD | D | I | A | TA |
| 07. I have a good relationship with my classmates. | *PH* | TD | D | I | A | TA |
| 08. I have time for extracurricular activities. | *TM* | TD | D | I | A | TA |
| 09. I don’t have enough free time. | *TM* | TD | D | I | A | TA |
| 10. My faith improves my quality of life. | *P* | TD | D | I | A | TA |
| 11. My life makes sense. | *P* | TD | D | I | A | TA |
| 12. My relationship with my teachers is good. | *LE* | TD | D | I | A | TA |
| 13. The contact with my patients increase my quality of life. | *LE* | TD | D | I | A | TA |
| 14. I push myself too much in my medical course. | *TM* | TD | D | I | A | TA |
| 15. I am pushed a lot by my teachers. | *TM* | TD | D | I | A | TA |
| 16. Don’t take care of my health. | *PH* | TD | D | I | A | TA |
| 17. I have time for cultural activities. | *TM* | TD | D | I | A | TA |
| 18. My medical course activities are hard for me. | *TM* | TD | D | I | A | TA |
| 19. I have enough time to study. | *TM* | TD | D | I | A | TA |
| 20. I have good access to medical care. | *LE* | TD | D | I | A | TA |
| 21. Most of my course classes are bad. | *LE* | TD | D | I | A | TA |
| 22. My relationship with my past year colleagues is good. | *LE* | TD | D | I | A | TA |
| 23. I can manage my time well. | *TM* | TD | D | I | A | TA |
| 24. My health is good. | *PH* | TD | D | I | A | TA |
| 25. My quality of life at the medical course is good. | *LE* | TD | D | I | A | TA |
| 26. My medical course deprives me of some personal appointments. | *TM* | TD | D | I | A | TA |
| 27. I have been feeling down lately. | *P* | TD | D | I | A | TA |
| 28. I have a good access to psychological care. | *LE* | TD | D | I | A | TA |
| 29. I have enough sleeping time. | *TM* | TD | D | I | A | TA |
| 30. I am satisfied with my undergraduate course. | *LE* | TD | D | I | A | TA |
| 31. I have time for my friends. | *TM* | TD | D | I | A | TA |
| 32. I can’t take care of my looks. | *PH* | TD | D | I | A | TA |
| 33. My Family expectation towards my performance decreases my quality of life. | *P* | TD | D | I | A | TA |
| 34. I have been feeling anxious lately. | *P* | TD | D | I | A | TA |
| 35. My university environment is competitive. | *LE* | TD | D | I | A | TA |
| 36. I am happy about my love life. | *P* | TD | D | I | A | TA |
| 37. My own expectation worsens my quality of life. | *P* | TD | D | I | A | TA |
| 38. My vitality is enough to do my activities in the medical course. | *P* | TD | D | I | A | TA |
| 39. I can’t absorb the content. | *P* | TD | D | I | A | TA |
| 40. I can properly eat. | *PH* | TD | D | I | A | TA |
| 41. I regularly do physical activities. | *PH* | TD | D | I | A | TA |
| 42. I haven’t been able to properly concentrate lately. | *P* | TD | D | I | A | TA |
| 43. I get stressed in my medical course. | *P* | TD | D | I | A | TA |
| 44. I am satisfied with my housing conditions. | *PH* | TD | D | I | A | TA |
| 45. I feel under pressure by having to financially depend on my family. | *LE* | TD | D | I | A | TA |
